# Targeting impulsivity in Parkinson’s disease using atomoxetine

**DOI:** 10.1093/brain/awu117

**Published:** 2014-06-03

**Authors:** Angie A. Kehagia, Charlotte R. Housden, Ralf Regenthal, Roger A. Barker, Ulrich Müller, James Rowe, Barbara J. Sahakian, Trevor W. Robbins

**Affiliations:** 1 Department of Neuroimaging, Institute of Psychiatry, King's College London, London SE5 8AF, UK; 2 Behavioural and Clinical Neuroscience Institute, University of Cambridge, Cambridge, UK; 3 Cambridge Cognition Limited, Cambridge, UK; 4 Department of Psychiatry, University of Cambridge, Cambridge, UK; 5 Division of Clinical Pharmacology, Rudolf-Boehm-Institute of Pharmacology and Toxicology, University of Leipzig, Germany; 6 Department of Clinical Neurosciences, University of Cambridge, Cambridge, UK; 7 Adult ADHD Service, Cambridgeshire and Peterborough NHS Foundation Trust, UK; 8 MRC Cognition and Brain Sciences Unit, Cambridge, UK; 9 Department of Psychology, University of Cambridge, Cambridge, UK

## Abstract

Noradrenergic dysfunction may play a significant role in cognition in Parkinson’s disease due to the early degeneration of the locus coeruleus. Converging evidence from patient and animal studies points to the role of noradrenaline in dopaminergically insensitive aspects of the parkinsonian dysexecutive syndrome, yet the direct effects of noradrenergic enhancement have not to date been addressed. Our aim was to directly investigate these, focusing on impulsivity during response inhibition and decision making. To this end, we administered 40 mg atomoxetine, a selective noradrenaline re-uptake inhibitor to 25 patients with Parkinson’s disease (12 female /13 male; 64.4 ± 6.9 years old) in a double blind, randomized, placebo controlled design. Patients completed an extensive battery of neuropsychological tests addressing response inhibition, decision-making, attention, planning and verbal short term memory. Atomoxetine improved stopping accuracy on the Stop Signal Task [*F*(1,19) = 4.51, *P = *0.047] and reduced reflection impulsivity [*F*(1,9) = 7.86, *P = *0.02] and risk taking [*F*(1,9) = 9.2, *P = *0.01] in the context of gambling. The drug also conferred effects on performance as a function of its measured blood plasma concentration: it reduced reflection impulsivity during information sampling [adjusted R^2 ^= 0.23, *F*(1,16) = 5.83, *P = *0.03] and improved problem solving on the One Touch Stockings of Cambridge [adjusted R^2 ^= 0.29, *F*(1,17) = 8.34, *P = *0.01]. It also enhanced target sensitivity during sustained attention [*F*(1,9) = 5.33, *P = *0.046]. The results of this exploratory study represent the basis of specific predictions in future investigations on the effects of atomoxetine in Parkinson’s disease and support the hypothesis that targeting noradrenergic dysfunction may represent a new parallel avenue of therapy in some of the cognitive and behavioural deficits seen in the disorder.

## Abstract (precis)

In a double-blind randomized placebo-controlled study, Kehagia *et al*. investigate the effects of a single dose of atomoxetine, a selective noradrenaline reuptake inhibitor, in 25 patients with Parkinson’s disease. Consistent with the presence of a longstanding noradrenergic deficit, atomoxetine improved stopping accuracy, and reduced reflection impulsivity during decision making.

## Introduction

Idiopathic Parkinson’s disease is characterized by progressive brain pathology affecting multiple neurotransmitter systems, leading to a dynamic and varied profile of physical, motor, cognitive and psychiatric dysfunction (Kehagia *et al.*, 2010*a*). At clinical onset, patients present with unilateral motor deficits largely reflecting dopaminergic and cholinergic dysfunction due to degenerative events in the substantia nigra and midbrain nuclei commencing up to 5 years earlier (Braak and Braak, 2000; Braak *et al.*, 2002). Dopaminergic replacement therapies in the form of the dopamine precursor l-DOPA as well as dopamine agonists and monoamine oxidase inhibitors aim at restoring striatal dopaminergic tone to alleviate the movement disorder. Psychopharmacological studies have thus focused on dopamine, and acute withdrawal studies have correspondingly shown that dopaminergic replacement therapies improves cognition reliant on dorsal fronto-striatal function, such as working memory, planning and attentional selection (Lange *et al.*, 1992; Cools *et al.*, 2001). Increases in impulsivity and deficits in learning may also ensue from dopaminergic enhancement, due to hypothetical overdosing of ventral cortico-striatal circuits, which are relatively intact in early Parkinson’s disease (Gotham *et al.*, 1988; Fern-Pollak *et al.*, 2004; Cools *et al.*, 2007).

The dopaminergic pathology with which the disease is mainly associated is, however, predated by other significant pathological events: Lewy bodies, or abnormal cytoplasmic inclusions, form in the locus coeruleus and lateral tegmental area (Cash *et al.*, 1987; Chan-Palay and Asan, 1989; Braak *et al.*, 1995; Zarow *et al.*, 2003), compromising noradrenergic neurotransmission throughout the cortex (Scatton *et al.*, 1983) up to a decade or longer before the motor dysfunction and ensuing Parkinson’s disease diagnosis (Hawkes *et al.*, 2010). As the largest group of noradrenergic neurons, the locus coeruleus is the main source of noradrenergic innervation to the neocortex, hippocampus and cerebellum (Moore and Bloom, 1979). This early noradrenergic hallmark manifests prodromally as a host of non-motor symptoms including sleep and mood disturbance (Remy *et al.*, 2005; Ishihara-Paul *et al.*, 2008; Alonso *et al.*, 2009; Chaudhuri and Odin, 2010) consistent with the role of the locus coeruleus in the regulation of these functions. To date, the impact of this pathological process, and noradrenergic therapy, on parkinsonian cognition has not been systematically investigated.

Given the central role of noradrenaline in attention, learning and executive functions (Chamberlain and Robbins, 2013), we have argued for the importance of examining noradrenergic contributions to cognition in Parkinson’s disease. Specifically, we have suggested that aspects of the Parkinson’s disease dysexecutive syndrome may also reflect this longstanding noradrenergic deficit (Kehagia *et al.*, 2009, 2010*a*, *b*). In this study, we focus primarily on impulsivity during response inhibition and decision-making.

As a multifaceted concept, impulsivity characterizes a range of behaviours that are ‘poorly conceived, prematurely expressed, unduly risky, or inappropriate to the situation and often result in undesirable outcomes’ (Daruna and Barnes, 1993). A minority of patients develop clinically significant impulsive compulsive behaviours or impulse control disorder, in the form of motor stereotypies such as punding, appetitive behaviours including hypersexuality and pathological gambling (Weintraub *et al.*, 2010*a*), as well as the compulsive use of excessive dopaminergic replacement therapies (Lawrence *et al.*, 2003). Impulse control disorder presents in a variety of conditions treated with dopamine agonists, such as restless leg syndrome (Cornelius *et al.*, 2010); in Parkinson’s disease, these agents increase the risk of impulse control disorder expression (Weintraub *et al.*, 2006) but they do not unequivocally cause it (Evans *et al.*, 2005; Voon *et al.*, 2007). Instead, individual differences such as novelty seeking, age at onset, a family history of gambling, alcohol use, depressive symptomology, as well as differences in underlying disease pathophysiology, particularly in ventral corticostriatal circuits (van Eimeren *et al.*, 2010), collectively render a patient vulnerable to the development of the disorder (reviewed in Cilia and van Eimeren, 2011).

In contrast to these reward-related aspects of impulsivity that reflect dopaminergic dysfunction in the small group of patients with Parkinson’s disease with impulse control disorder, impulsive behaviour unaffected by dopaminergic manipulations is frequently revealed in the course of assessing patients with Parkinson’s disease without impulse control disorder using a range of tasks probing different facets of the construct: response inhibition, reflection impulsivity, delay discounting, and delay aversion rely on different neurobiological substrates in terms of underlying neurochemistry and circuitry (Evenden, 1999; Robbins and Arnsten, 2009). It is these aspects of impulsivity we focus on here. For example, patients with Parkinson’s disease show deficits on the Stop Signal Task unrelated to general slowing and global cognitive impairment (Gauggel *et al.*, 2004; Obeso *et al.*, 2011*a*), as well as other tasks indexing inhibition, such as the go/no-go (Cooper *et al.*, 1994; Beste *et al.*, 2010; Baglio *et al.*, 2011), anti-saccade (Rivaud-Pechoux *et al.*, 2007), flanker (Praamstra and Plat, 2001; Wylie *et al.*, 2005, 2009), Hayling (Bouquet *et al.*, 2003) and random number generation (Obeso *et al.*, 2011*a*). Commensurate with the significant non-dopaminergic pathology caused by Parkinson’s disease, acute dopaminergic withdrawal studies have gone some way in disambiguating medication from disease effects, by highlighting a range of impulsive behaviours that seem insensitive to dopaminergic status. Patients with Parkinson’s disease show longer stop signal reaction time both ON and OFF dopaminergic medication compared with healthy control subjects (Obeso *et al.*, 2011*b*), consistent with animal work showing that blocking the re-uptake of dopamine (Bari *et al.*, 2009) or increasing its synthesis by l-DOPA administration (Overtoom *et al.*, 2003) has no effect on stop signal reaction time.

In humans, enhancing noradrenaline neurotransmission using the selective noradrenaline re-uptake inhibitor atomoxetine improves stop signal reaction time in healthy individuals (Chamberlain *et al.*, 2006) as well as in adult patients with attention deficit hyperactivity disorder (Chamberlain *et al.*, 2007), who exhibit response inhibition deficits and in whom the drug is licensed for clinical use. In the rat, atomoxetine has been shown to enhance inhibition on the stop signal task, as well as the five-choice serial reaction time and delay discounting tasks (Robinson *et al.*, 2008). Its efficacy in ameliorating impulsivity in high impulsive rats has also been replicated in an animal model of attention deficit hyperactivity disorder (Fernando *et al.*, 2012). Atomoxetine inhibits noradrenaline reuptake through the noradrenaline transporter in the prefrontal cortex (Bymaster *et al.*, 2002), and increases the phasic-to-tonic ratio of evoked responses in the locus coeruleus (Bari and Aston-Jones, 2013). Beyond its main noradrenergic character, atomoxetine also exerts glutamatergic effects by antagonizing the *N*-methyl-d-aspartate receptor (Ludolph *et al.*, 2010), and enhances extracellular prefrontal dopamine levels for which the noradrenaline transporter also has high affinity (Bymaster *et al.*, 2002).

To investigate the role of noradrenaline neurotransmission in cognitive deficits in Parkinson’s disease and highlight its role in response inhibition and reflection impulsivity in this group, we administered a single dose of atomoxetine in a double-blind randomized placebo controlled design. Given the presence of noradrenergic dysfunction in Parkinson’s disease, and the close link between noradrenaline and impulsivity, a drug such as atomoxetine with predominantly noradrenergic action and extensive evidence of effects on impulsivity is an ideal candidate. Only two studies to date have addressed its effects in Parkinson’s disease. An 8-week open label flexible dose trial in 12 patients reported improvements in overall executive function as assessed by the Frontal Systems Behavioural Scale and the Connors Adult Attention Deficit Hyperactivity Disorder Rating Scale (Marsh *et al.*, 2009). Another study, assessing its efficacy in improving neuropsychiatric symptoms in Parkinson’s disease, found reductions in daytime somnolence and improved global cognition as assessed by the Mini-Mental State Examination, but no mood effect (Weintraub *et al.*, 2010*b*). Apart from manipulating dopaminergic therapy, which can be detrimental to motor symptoms, there are currently no pharmacological treatments for impulsivity in Parkinson’s disease. This study is the first to investigate the noradrenergic hypothesis concerning diverse yet specific facets of impulsive behaviour seen in Parkinson’s disease.

## Methods and materials

### Patients

Twenty-five participants (12 female and 13 male) were recruited through the John van Geest Brain Repair Centre, Parkinson’s disease Research Clinic, University of Cambridge. Idiopathic Parkinson’s disease was diagnosed according to UK Parkinson’s Disease Society Brain Bank criteria. Exclusion criteria were: a history of other significant neurological disorder; stroke or brain damage; current psychiatric comorbidity; noradrenergic medications; uncontrolled hypertension; colour blindness; glaucoma; Mini-Mental State Examination score <23 at earlier assessment.

### Pharmacotherapy

Twenty-two patients were treated with levodopa, and of these patients, nine were receiving the *N*-methyl-d-aspartate antagonist amantadine and eight were receiving a catechol-o-methyl transferase inhibitor. The majority of patients (21 of 25) were also medicated with dopamine agonists: the mixed D2, D3, D4 agonist ropinirole (10 patients), or the D2, D3 agonist pramipexole (11 patients). Three of these patients were on agonist monotherapy, using only ropinirole (one patient) or pramipexole (two patients). Further details of individual daily drug regimes can be found in the Supplementary material. As atomoxetine would only be used clinically as an adjunctive treatment, all participants remained on their current medications for the duration of the study. They were screened for impulse control disorder with the South Oaks Gambling Screen (Lesieur and Blume, 1987), the Mini-International Neuropsychiatric Interview (Sheehan *et al.*, 1998) and the Minnesota Impulse Disorders Interview (Christenson *et al.*, 1994). No behaviours that were indicative of an impulse control disorder were recorded. Six patients reported past visual hallucinations, which had disappeared after their medication was adjusted. Average levodopa equivalent daily dose, demographics and patient characteristics such as IQ as indexed by the Wechsler Test of Adult Reading (Wechsler, 1981) are presented in Table 1. Levodopa equivalent daily dose was calculated by taking into account the full pharmacotherapeutic regime based on theoretical equivalence. The study was approved by the Cambridge Local Research Ethics Committee (09/H0302/84) and performed in accordance with the ethical standards laid down in the 1964 Declaration of Helsinki. All participants gave informed consent prior to participation.

**Table 1:** Demographic and clinical characteristics of the two patient randomization groups

	Atomoxetine/placebo group (*n = *13)	Placebo/atomoxetine group (*n = *12)
Age, years	64.8 (8.5)	64.1 (5.3)
Education, years	14.3 (3.2)	14.6 (2.5)
Mini-Mental State Examination	28.6 (0.96)	28.8 (13)
IQ	105.3 (8.9)	106.7 (6.2)
Unified Parkinson’s Disease Rating Scale (motor)	26.4 (13.7)	17.2 (13.5)
Total LEDD mg/d	1010.4 (524.5)	1311.5 (741.5)
Dopamine agonist LEDD mg/d	248.8 (44.8)	223.1 (54.7)
Beck Depression Inventory	7.8 (4.22)	7.2 (4.2)
Epworth Sleepiness Scale	9.8 (4.47)	11.1 (4)
Verbal fluency	51.1 (16.6)	54 (11.8)
Semantic fluency	19.8 (3.5)	21.33 (5.2)
State and Trait Anxiety Inventory: state	12.4 (6.6)	9.9 (8.1)
State and Trait Anxiety Inventory: trait	15.8 (6.1)	14.1 (11.3)

Data represent mean (SD) values. LEDD = levodopa equivalent daily dose. There were no significant differences.

### Design

The design was crossover, double-blind, placebo-controlled, with 12 patients randomized to receive a single oral dose of a lactose placebo on the first session followed by 40 mg of atomoxetine on the second session (placebo/atomoxetine group) and 13 randomized to receive atomoxetine first (atomoxetine/placebo group). Testing sessions were separated by at least 5 days [mean = 10.2, standard deviation (SD) = 4.6], but not longer than 3 weeks to ensure there were no changes in disease severity or concurrent medication. The randomization groups were matched for age, IQ, education level, disease severity as indexed by the Unified Parkinson’s Disease Rating Scale motor subscale (Fahn *et al.*, 1987), total levodopa equivalent daily dose as well as dopamine agonist levodopa equivalent daily dose (Table 1). A dose of 40 mg was used to ensure tolerability based on previous studies (Jankovic, 2009; Marsh *et al.*, 2009; Weintraub *et al.*, 2010*b*). As peak plasma concentration for atomoxetine is achieved ∼1–2 h after oral dosing in healthy adults (Sauer *et al.*, 2005), testing commenced 1.5 h after administration and lasted ∼2.5 h.

### Samples and measures

Blood pressure and pulse measurements were taken at three time points: before drug administration, immediately before testing (1.5 h post-drug), and on completion of the study (4 h post-drug). Blood samples were taken immediately before testing (1.5 h post-drug), and on completion of the study (4 h post-drug), and were used to estimate the mean drug plasma concentration for each participant for each session. Patients completed the State and Trait Anxiety Inventory (Spielberger *et al.*, 1983), Epworth Sleepiness Scale (Johns, 1991), Beck Depression Inventory (Beck *et al.*, 1961) and verbal (FAS) and semantic (animals) fluency (Benton, 1968). They also completed visual analogue scales (Bond and Lader, 1974) to rate their experience in terms of 16 dimensions at these intervals during the session: immediately before drug administration, halfway through the cognitive testing session, and on completion of testing. The extreme points of each dimension: alert–drowsy, calm–excited, strong–feeble, muzzy–clear headed, well-coordinated–clumsy, lethargic–energetic, contented–discontented, troubled–tranquil, mentally slow–quick witted, tense–relaxed, attentive–dreamy, incompetent–proficient, happy–sad, antagonistic–amicable, interested–bored, and withdrawn–gregarious were separated by 10-cm lines, and subjects marked where, on each line, they felt they ranked. Alertness and tranquillity factors were calculated (Bond and Lader, 1974; Herbert *et al.*, 1976).

### Cognitive assessment

The neuropsychological test battery included paper and pencil assessments and tests from the Cambridge Neuropsychological Test Automated Battery (CANTAB) (www.camcog.com), which were administered in the same order on both sessions. They are described briefly here; the reader is directed to the cited references for more details. Three tasks measured different forms of impulsivity. On the Stop Signal Task of response inhibition (Logan *et al.*, 1984), participants make speeded left or right responses on go trials but withhold their response on stop trials (signalled by a 300-Hz tone). A tracking algorithm generates the stop signal delay, which varies on each trial so that the subject succeeds at inhibiting a prepared response on overall half the trials (Band *et al.*, 2003). The stop signal reaction time reflects differences in stop signal delay, at a success rate of 50%, once differences in go reaction time are factored out. The race model allows estimation of the time required to suppress a go response (stop signal reaction time). In the Cambridge Gamble Task (Rogers *et al.*, 1999) participants decide whether a randomly hidden token is more likely to be in a red or blue box within a display of 10 boxes (the ratio of which varies within a display of 10 boxes), and place bets (in ascending and descending order) on their choice being correct. Deliberation time, delay aversion (the difference in risk taking between ascending and descending conditions) and risk adjustment (the rate at which subjects vary risk taking in response to the ratio of red to blue boxes on each trial) are the principal variables of interest. In the Information Sampling Task (Clark *et al.*, 2006), which measures reflection impulsivity, participants are presented with a 5 × 5 matrix of grey boxes that reveal one of two colours when they are touched. The participant has to decide which colour is in the majority by opening as many boxes as they like in order to win points. In the fixed condition, 100 points are given for a correct response, in the descending condition, 250 points are available to begin with, which decrease by 10 for every box opened. The administration order of the parallel versions of the Cambridge Gamble Task and Information Sampling Task (ascend and descend; fixed win and decreasing win) was counter-balanced across the atomoxetine/placebo and placebo/atomoxetine groups.

In addition to the impulsivity measures, the Rapid Visual Processing test of sustained attention (Coull *et al.*, 1995) was administered. In this task, participants must detect target sequences (e.g. 2-4-6) of digits as they are sequentially presented at a rate of 100/min. Planning and problem solving was assessed using the One Touch Stockings of Cambridge, a variant of the Tower of London (Owen *et al.*, 1995), where participants indicate the minimum number of moves required to solve a problem by a single touch-screen response. Verbal working memory was assessed with the Forward and Backward Digit Span from the Wechsler Adult Intelligence Scale (Wechsler, 1981). All computerized tasks were run on a Paceblade touch screen computer and responses registered via the touch-sensitive screen or a button box.

### Analyses

#### Blood biochemistry

Plasma levels of atomoxetine were analysed in all the pre- and post-session active treatment samples obtained, using a high performance liquid chromatographic method (Guo *et al.*, 2007) outlined in Chamberlain *et al.* (2009).

#### Neuropsychological results

The data were submitted to repeated-measures ANOVA with treatment (drug or placebo) as the within-subject factor and administration order (atomoxetine/placebo or placebo/atomoxetine) as the between subjects factor. Where the effect or interactions with administration order were significant, session-specific effects were addressed. Relationships between drug plasma concentration and performance changes (atomoxetine versus placebo) on each task were also examined. Shapiro-Wilk tests were performed to ensure normality across all measures and transforms were applied were necessary. Greenhouse-Geisser corrections were applied where the assumption of sphericity was violated. Bonferroni correction was not deemed appropriate given that the possibility of a type I error is less problematic than a type II error in a novel study, and that different but non-independent aspects of impulsivity were investigated. Analyses were performed using SPSS software version 15.

## Results

### Physiological effects

Variability in atomoxetine plasma concentration was large (range 45.3–723.8 ng/ml). Drug plasma levels increased from the first to the second sample in seven participants, and decreased in the remaining 18. Mean plasma levels of atomoxetine (average of pre- and post-testing values) were 308.9 ± 121.2 ng/ml (range 96.1–560.2) during active treatment (Table 2). Due to this large variability, data from two patients in whom the drug was not detectable in the first sample, and one with an anomalously low score (<100 ng/ml) were excluded.

**Table 2:** Atomoxetine plasma concentration

Participant	Sample 1	Sample 2	Mean
**1**	575.2	324.3	449.8
**2**	n.d	291.2	–
**3**	77.5	317.1	197.3
**4**	45.3	146.8	96.05
**5**	604.7	188.3	396.5
**6**	n.d	72.6	–
**7**	190.4	368.2	279.3
**8**	489.7	267.1	378.4
**9**	424	133.1	278.6
**10**	189.4	277.1	233.3
**11**	409.7	239	324.4
**12**	650	344.8	497.4
**13**	436.4	131.3	283.9
**14**	106.1	590.3	348.2
**15**	523.9	264.5	394.2
**16**	502.6	229.2	365.9
**17**	412.9	135	274
**18**	346	330.4	338.2
**19**	463.7	131.6	297.7
**20**	253	156.1	204.6
**21**	454.1	320.9	387.5
**22**	551	130.6	340.8
**23**	312.7	91.8	202.3
**24**	550.7	276.1	413.4
**25**	723.8	396.5	560.2

Plasma levels of atomoxetine are shown in ng/ml. Atomoxetine was not detected (n.d.) in the first sample for two participants. Sample 1 is the first blood sample collected on the active drug visit, at the start of the cognitive testing, 1.5 h after drug administration. Sample 2 is the second blood sample collected on the active drug visit, at the end of the testing session, ∼4 h after drug administration.

### Subjective effects

Atomoxetine was well tolerated. Unwanted effects on the drug visit included feeling more emotional (*n = *2) and headache during the testing session (*n = *1) and raised blood pressure at the end of the testing session (*n = *1) on the placebo visit. Atomoxetine enhanced alertness [*F*(1,15) = 5.86, *P = *0.03], and the effect of time on increasing alertness was only seen when atomoxetine was administered first [time × order: *F*(1.52,22.82) = 5.82, *P = *0.01]: in these patients, atomoxetine increased alertness [*F*(1,9) = 8.19, *P = *0.02] as the session progressed [*F*(1.46,13.14) = 8.96, *P = *0.006] but there was no treatment × time interaction (*F* < 1). No effects were seen in the group receiving placebo first (*F* < 1). There were no effects on tranquillity.

### Neuropsychological effects

Scores for the behavioural measures in the atomoxetine and placebo conditions are presented in Table 3.

**Table 3:** Summary of behavioural measures

Measure	Atomoxetine		Placebo	
	Session 1	Session 2	Session 1	Session 2
**Stop Signal Task**				
Successful stops (%)	54.8 (2.1)	54.5 (2.2)	51.3 (2.9)	48 (2.8)
Median go RT (ms)	479 (35)	453 (37)	459 (24)	420 (23)
SSRT (ms)	254 (31)	241 (21)	210 (21)	225 (20)
SSD	231 (39)	218 (41)	235 (33)	168 (39)
**Cambridge Gamble Task**				
Deliberation time	3268 (287)	2426 (287)	2817 (248)	2609 (287)
Proportion bet	54.8 (4.5)	59 (4.5)	58.7 (4.8)	55.5 (4.8)
Risk adjustment	0.81 (0.28)	0.96 (0.28)	0.88 (0.27)	1.19 (0.27)
Delay aversion	0.28 (0.06)	0.19 (0.06)	0.24 (0.07)	0.26 (0.07)
**Information Sampling Task**				
Number of boxes opened	15.33 (2.27)	11.85 (2.41)	13.49 (2.54)	13.86 (2.39)
Box opening latency (ms)	1348 (185)	1161 (196)	1018 (185)	1265 (174)
Decision latency (ms)	23385 (2546)	14420 (2701)	14952 (2969)	19387 (2799)
**One-Touch Stockings of Cambridge**				
Problems solved on first choice	3.11 (0.13)	3.34 (0.14)	3.1 (0.15)	3.27 (0.14)
Latency to first choice (ms)	17559 (1639)	17116 (1719)	19754 (2034)	15037 (1940)
Latency to correct (ms)	21544 (2071)	20657 (2172)	27555 (3451)	17983 (3291)
**Rapid Visual Information Processing**				
Mean latency (ms)	483 (38)	473 (41)	540 (50)	487 (46)
Hits	14.25 (1.71)	16 (1.87)	13.5 (2.11)	15.25 (1.93)
False alarms	3.33 (1.03)	3.8 (1.13)	5.8 (2)	3.08 (1.82)
A’	0.87 (0.02)	0.89 (0.02)	0.86 (0.02)	0.88 (0.02)
B’	0.88 (0.03)	0.88 (0.03)	0.84 (0.05)	0.88 (0.05)
**Digit Span**				
Forward	10.22 (0.75)	8.75 (0.79)	8.88 (0.7)	9.78 (0.66)
Backward	7.33 (0.93)	6.63 (0.98)	6.63 (0.83)	7.56 (0.78)

Data represent mean raw values (SEM). RT = reaction time; SSRT = stop signal reaction time; SSD = stop signal delay.

### Stop Signal Task

**Figure 1:**
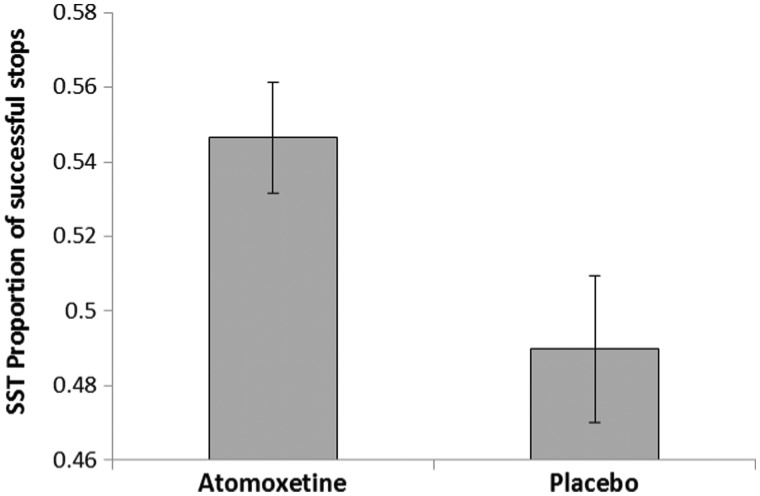
Effect of atomoxetine on the Stop Signal Task (SST). Patients tested on atomoxetine exhibited a greater proportion of successfully inhibited responses. Error bars represent standard errors.

Twenty-one data sets were analysed as one participant did not complete the Stop Signal Task. Atomoxetine conferred a significant increase in the proportion of successful stops on both test days [*F*(1,19) = 4.51, *P = *0.047] (Fig. 1). Although the drug did not significantly increase go reaction time [*F*(1,19) = 3.02, *P = *0.1], there was a significant interaction with order [drug × order: *F*(1,19) = 4.52, *P = *0.047] indicating longer go reaction time on the first [*F*(1,10) = 4.81, *P = *0.05] but not the second session (*F* < 1). The effects for stop signal delay were all at trend level: the treatment × order interaction [*F*(1,19) = 3.26, *P = *0.087] indicated longer stop signal delay on the first [*F*(1,10) = 3.98, *P = *0.07] but not on the second session (*F* < 1). Given the differences in successful inhibition, the integration method (Verbruggen and Logan, 2009) was used to calculate stop signal reaction time. One outlier (578 ms, mean = 247, SD = 100) was excluded. There were no effects of treatment or order (both F < 1), nor did these factors interact [*F*(1,18) = 2.03, *P = *0.17]. The relationship between atomoxetine plasma concentration and stop signal reaction time did not reach significance [R^2 ^= 0.16, adjusted R^2 ^= 0.11, *F*(1,18) = 3.34, *P = *0.08].

### Cambridge Gamble Task

**Figure 2:**
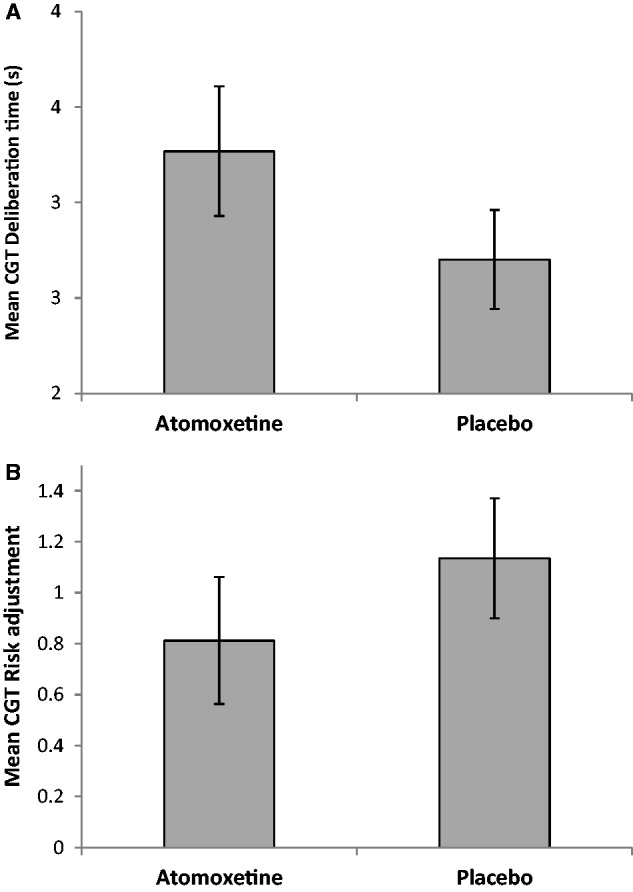
Effects of atomoxetine on the Cambridge Gamble Task. Atomoxetine reduced impulsivity when it was administered on the first session. Patients receiving atomoxetine exhibited (**A**) increased deliberation time and (**B**) more modest increases in betting as the probability of winning increased. Error bars represent standard errors.

As two participants did not complete the Cambridge Gamble Task, 20 data sets were analysed. There were no effects of treatment [*F*(1,18) = 1.14, *P = *0.3] or order [*F*(1,18) = 2.1, *P = *0.16] on deliberation time, but these factors interacted [*F*(1,18) = 6.38, *P = *0.02]: atomoxetine increased deliberation time on the first session [*F*(1,9) = 7.86, *P = *0.02] (Fig. 2A) but not on the second (*F* < 1). The pattern of results for risk adjustment was similar. There were no effects of treatment [*F*(1,18) = 2.62, *P = *0.12] or order (*F* < 1), but there was a significant interaction [*F*(1,18) = 6.08, *P = *0.02]: patients on atomoxetine exhibited smaller modulations in risk taking in response to more favourable box ratios on the first [*F*(1,9) = 9.2, *P = *0.01] (Fig. 2B) but not the second session (*F* < 1).

### Information Sampling Task

**Figure 3:**
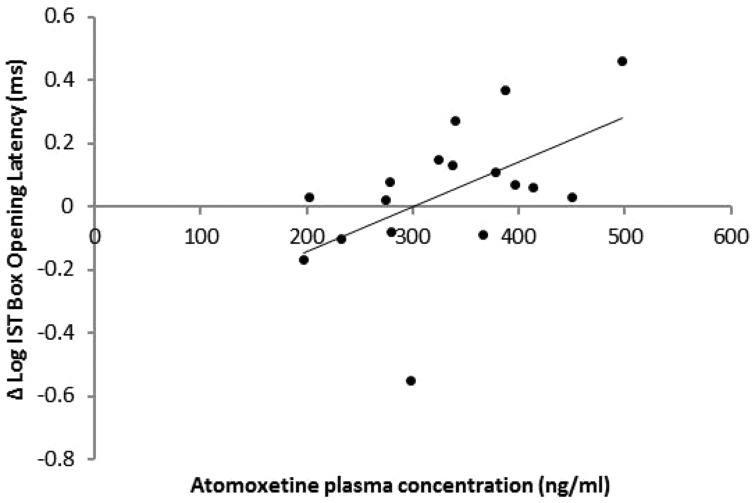
Effect of atomoxetine plasma concentration on mean box opening latency in the fixed win condition of the Information Sampling Task (IST). Plasma atomoxetine levels predicted increases in box opening latency when patients were tested on drug compared to placebo.

Data from 17 patients were analysed. For mean colour decision latency, one data point (39.29 s) was excluded as an outlier (group mean = 16.85 s, SD = 8.41). There were no effects of treatment [*F*(1,14) = 2.64, *P = *0.13] or order [*F*(1,14) = 2.16, *P = *0.16] but the trend for an interaction [*F*(1,14) = 4.19, *P = *0.06] indicated that atomoxetine increased decision latency compared to placebo when it was administered on the first [*F*(1,8) = 4.54, *P = *0.05], but not the second session (*F* < 1). Atomoxetine plasma concentration predicted increases in box opening latency [R^2 ^= 0.28, adjusted R^2 ^= 0.23, *F*(1,16) = 5.83, *P = *0.03] indicating reductions in reflection impulsivity (Fig. 3).

#### Rapid visual information processing

Twenty-two data sets were analysed. There were no effects of treatment or order on any measure. There was no effect of treatment on mean log latency [*F*(1,20) = 3.13, *P = *0.09] but there was an interaction with order [*F*(1,20) = 4.72, *P = *0.04], and a further treatment × order trend was seen for correct signal detection A’ [*F*(1,20) = 3.98, *P = *0.06], indicating significant improvements when atomoxetine was administered on the second session for mean log latency [*F*(1,9) = 6.87, *P = *0.028] and A’ [*F*(1,9) = 5.33, *P = *0.046]. There were no treatment effects when atomoxetine was administered on the first session (all *F* < 1).

### One Touch Stockings of Cambridge

**Figure 4:**
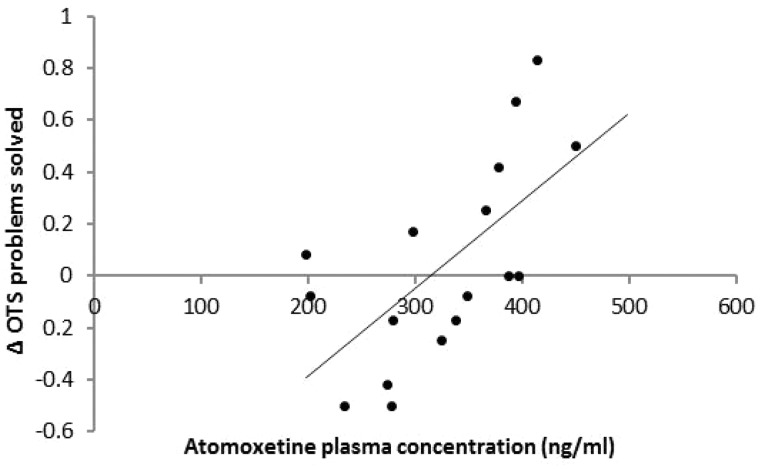
Performance on the One-Touch Stockings (OTS) of Cambridge. Atomoxetine plasma concentration predicted superior problem solving.

Data sets from 21 patients were analysed. There were no effects of treatment or order on any measure. The treatment × administration order interaction for latency to first choice [*F*(1,19) = 5.28, *P = *0.03] signified practice effects from the first to the second session. Atomoxetine plasma concentration predicted superior performance seen on the drug compared with placebo in terms of the total number of problems solved [R^2 ^= 0.33, adjusted R^2 ^= 0.29, *F*(1,17) = 8.34, *P = *0.01] (Fig. 4).

### Digit Span

No effects were seen for forward or backward Digit Span (all *F* < 1).

## Discussion

This is the first comprehensive investigation of the effects of the selective noradrenaline reuptake inhibitor atomoxetine on response inhibition and reflection impulsivity in Parkinson’s disease. We used atomoxetine to test the hypothesis that acute noradrenergic augmentation in Parkinson’s disease would confer benefits to dopaminergically insensitive aspects of the dysexecutive syndrome which hypothetically reflect the presence of significant, parallel but as yet understudied noradrenergic dysfunction.

The emergent picture from this exploratory study suggests that atomoxetine may enhance inhibition and lead to a more conservative behavioural profile. Patients were more successful at inhibiting responses on atomoxetine, showed longer deliberation times and more conservative bets in response to improved odds of winning, and exhibited a more subtle but consistent reduction in reflection impulsivity during information sampling. Crucially, these effects were not the result of sedation, as the drug significantly enhanced subjective ratings of alertness. Moreover, atomoxetine improved sustained attention leading to faster responses and improving target detection on the second session. An improvement in abstract problem solving as a function of its plasma concentration was also observed. This pattern of results represents a starting point for the formation of concrete hypotheses concerning the effects of atomoxetine on specific aspects of cognition in Parkinson’s disease, to be directly investigated in future studies.

The first notable finding is the effect of atomoxetine on the proportion of successful stops on the Stop Signal Task. Previous studies comparing patients with Parkinson's disease to controls demonstrated longer stop signal reaction (Gauggel *et al.*, 2004; Obeso *et al.*, 2011a) and no effects of dopaminergic medication on any Stop Signal Task measure (Obeso *et al.*, 2011b; Alegre *et al.*, 2013). To our knowledge, this is the first observation of an improvement in inhibitory success on the Stop Signal Task following atomoxetine, in healthy or patient groups, but no stop signal reaction time benefit, contrary to previous findings of stop signal reaction time effects in both healthy (Chamberlain *et al.*, 2006) and attention deficit hyperactivity disorder cohorts (Chamberlain *et al.*, 2007). In Parkinson’s disease, atomoxetine led to a shift to a more conservative response strategy, so that patients favoured stopping accuracy over speed, despite the tracking function and experimental instructions (Sylwan, 2004; Wostmann *et al.*, 2013). This pattern of behaviour on the Stop Signal Task suggests that future investigations should focus less on reactive, motor-specific processes *per se*, but rather on biasing competitive interactions between proactive and reactive processes at the superordinate executive level.

Evidence from neuropsychological studies (Aron *et al.*, 2003*a*; Rieger *et al.*, 2003; Floden and Stuss, 2006), neuroimaging (Rubia *et al.*, 2001; Aron *et al.*, 2003*b*; Nachev *et al.*, 2008; Pauls *et al.*, 2012) and deep brain stimulation (Jahanshahi *et al.*, 2000; van den Wildenberg *et al.*, 2006; Ballanger *et al.*, 2009; Alegre *et al.*, 2013; Favre *et al.*, 2013) has led to a broad functional characterization of a cortico-subcortical network involved in reactive inhibition which includes the inferior and orbital frontal gyrus, pre-supplementary motor area and insula, as well as the subthalamic nucleus. However, in understanding impulsivity, it is necessary to extend the concept of inhibition beyond the reactive, phasic mode and consider its tonic character. Because the mere presentation of a stimulus elicits transient automatic sensorimotor cortex activation (Jaffard *et al.*, 2007), proactive inhibition is normally applied to all prepotent responses in the face of uncertainty. Patients with Parkinson’s disease demonstrate disproportionate proactive inhibition (Favre *et al.*, 2013), which is normalized by subthalamic nucleus stimulation but not dopaminergic medication, pointing to the pivotal role of this structure in inhibition as well as to the non-dopaminergic character of the deficit in Parkinson’s disease. The effects of noradrenergic enhancement on proactive inhibition in Parkinson’s disease are a clear target for future investigation.

Intriguingly, lesioning the subthalamic nucleus in the rat speeds up go reaction time and impairs stopping accuracy (Baunez *et al.*, 1995), rendering the animal more impulsive by disinhibiting basal ganglia outflow, conferring the exact opposite effects to those we report following the administration of atomoxetine. Conversely, atomoxetine increases blood oxygen level-dependent activity in the subthalamic nucleus and thalamus in the rat (Easton *et al.*, 2007). Notwithstanding the unknown effects of atomoxetine on a compromised cortex and locus coeruleus, atomoxetine may enhance inhibition in Parkinson’s disease through the subthalamic nucleus. The effect may be mediated by: (i) enhancing prefrontal noradrenaline, and, in cognitive terms, top–down control; and (ii) decreasing tonic spiking in the locus coeruleus and affecting corticocoeruleal coherence in circuits that include the subthalamic nucleus (Bari and Aston-Jones, 2013).

The reductions in risk taking and reflection impulsivity seen on the gambling and information sampling tasks collectively also indicate a shift to more conservative, deliberative behaviour. These particular effects were weaker, emerging when the drug was administered on the first session, when the patients were task naïve; we hypothesize that the effect of atomoxetine on the second session is counteracted by the effect of practice, which reduces reflection time. Nonetheless, findings on these tasks are important in validating the choice of atomoxetine in probing noradrenaline but not dopamine-dependent aspects of impulsivity. Although atomoxetine enhances prefrontal dopamine (Bymaster *et al.*, 2002; Swanson *et al.*, 2006), its impact on dopaminergic transmission in medicated Parkinson’s disease remains unknown. In this study, atomoxetine improved reflection impulsivity, and had no discernible effects on dopaminergically sensitive measures on these tasks related to reward sensitivity and the probability of winning, theoretically vulnerable to overdosing by further dopaminergic augmentation. As discussed, dopamine agonists can have deleterious effects on decision making in the face of uncertainty and reward in Parkinson’s disease by disrupting reward prediction error, or learning from losing (van Eimeren *et al.*, 2009). Moreover, this study focused on the role of noradrenaline in impulsivity in Parkinson’s disease, so we sought to avoid confounds by excluding patients with impulse control disorder. The incidence of impulse control disorder in the Parkinson’s disease population has been estimated at 13.6% (Weintraub *et al.*, 2010*a*), and as discussed dopamine agonists are one of the major risk factors. However, the proportion of patients treated with dopamine agonists by far exceeds those who develop an impulse control disorder. In the current study, although the majority of patients were medicated with a dopamine agonist, none exhibited such behaviours before or at the time of testing, and no differences at placebo baseline were revealed by a *post hoc* comparison between the agonist treated (*n = *19) and agonist naïve (*n = *4) patients in the current sample (Supplementary material). We acknowledge that it is impossible to rule out the possibility of the future emergence of impulse control disorder in any of the individuals tested. Future studies could directly address this issue by including longitudinal follow up and investigating these effects in agonist naive patients.

The other notable anti-impulsivity agent used in attention deficit hyperactivity disorder, methylphenidate, which has a primarily dopaminergic impact but also blocks the dopamine and noradrenaline transporters presynaptically and affects subcortical dopamine mechanisms (Volkow *et al.*, 2001), has subtly different effects in Parkinson’s disease compared to those we report here on atomoxetine. In Parkinson’s disease, methylphenidate was shown to reduce apathy (Chatterjee and Fahn, 2002; Moreau *et al.*, 2012) and daytime sleepiness (Devos *et al.*, 2007; Moreau *et al.*, 2012) presumably reflecting its noradrenalinergic impact (although dopaminergic effects cannot be discounted; del Campo *et al.*, 2013). It improved attention on the Mindstreams test battery (Auriel *et al.*, 2006), but led to reaction time inflations on a choice reaction time task (Devos *et al.*, 2007). Its effects on impulsivity in Parkinson’s disease have not to date been examined, possibly also because unlike atomoxetine (Upadhyaya *et al.*, 2013), methylphenidate has high abuse potential (Kollins *et al.*, 2001).

The attentional enhancement observed on the sustained attention task could be invoked as an alternative interpretation for the aforementioned effects on inhibition. This second session effect demonstrated here in patients with Parkinson’s disease replicates that previously reported in adult attention deficit hyperactivity disorder patients (Turner *et al.*, 2004) and young healthy volunteers (Crockett *et al.*, 2010), and appears to be specific to the action of atomoxetine, as methylphenidate only improves response latency (Elliott *et al.*, 1997). However, this account is unlikely because the drug improved inhibition on the Stop Signal Task across both sessions, but inflated go reaction time only on the first; moreover, putatively enhanced attention to the stop signal should affect stop signal reaction time, and this was not seen. Such attentional augmentation builds upon early work linking vigilance changes in Parkinson’s disease to altered noradrenaline metabolism (Stern *et al.*, 1984) and may point to the drug’s aforementioned direct effects on the locus coeruleus. The finding we report is clinically significant, particularly for patients suffering from non-motor symptoms including daytime somnolence, and in this case also atomoxetine’s attentional effects in Parkinson’s disease should be systematically investigated.

A final point concerns absorption and pharmacokinetics. Impaired gastrointestinal function and poor absorption in Parkinson’s disease has been causally linked to the troublesome ‘ON-OFF’ phenomenon and erratic plasma peaks of l-DOPA (Nutt *et al.*, 1984). High fat meals interfere with the absorption rate of atomoxetine (Christman *et al.*, 2004) and individual differences in atomoxetine pharmacokinetics have been demonstrated between extensive and poor metabolizers (Sauer *et al.*, 2003, 2005). In the current study, we saw considerable variability in atomoxetine plasma concentration, which could reflect any of the aforementioned issues. The 40 mg dose could be considered conservative, compared to studies in healthy subjects and adult patients with attention deficit hyperactivity disorder using doses up to 60 mg (Chamberlain *et al.*, 2006, 2007; Gilbert *et al.*, 2006) and 90 mg (Heil *et al.*, 2002). Future studies may opt for a higher or flexible dose, individually adjusted for each patient.

Collectively, we have interpreted these early findings on the effects of atomoxetine in Parkinson’s disease as pointing to a shift to a more conservative response strategy rather than a clear benefit. Yet these observations do not suggest regression to bradyphrenia (Wilson, 1954; Rogers *et al.*, 1987), historically associated with descriptions of the disease, because the drug (i) increased subjective ratings of alertness; (ii) conferred clear attentional benefits; and (iii) did not cause general slowing across tasks. The rationale for exploring the profile of atomoxetine in Parkinson’s disease and predicted benefits following noradrenergic enhancement were predicated on the known longstanding noradrenergic dysfunction originating in the early degenerative events affecting the locus coeruleus. Thus, these observations collectively represent a solid starting point for the development of specific hypotheses concerning the role of atomoxetine in non-motor symptoms in Parkinson’s disease.

## Supplementary Material

Supplementary Data
