# Supplementary material for: Targeting impulsivity in Parkinson’s disease using atomoxetine
Source: Brain. 2014 Jun 3;137(7):1986–97. doi: 10.1093/brain/awu117 (PMC4065022; doi:10.1093/brain/awu117)
Supplement: Supplementary Data [file supp_137_7_1986__index.html]

Targeting impulsivity in Parkinson’s disease using atomoxetine — Supplementary Data 

# Targeting impulsivity in Parkinson’s disease using atomoxetine

## Supplementary Data

files

**Files in this Data Supplement:**

- Supplementary Data - zip file
