# Supplementary material for: Targeting impulsivity in Parkinson’s disease using atomoxetine
Source: Brain. 2014 Jun 3;137(7):1986–97. doi: 10.1093/brain/awu117 (PMC4065022; doi:10.1093/brain/awu117)
Supplement: Supplementary Data [file supp_awu117_suppl_data.zip › brain-2013-02050-File010.docx]

Supplementary Table 1. Daily dose (mg) of levodopa and one of the two DA agonists, pramipexole or ropinirole, for each subject.

| **Subject** | **Levodopa** | **DA agonist** | |
| --- | --- | --- | --- |
|  |  | **Pramipexole** | **Ropinirole** |
| 1 | 500 | 0 | 0 |
| 2 | 800 | 2.1 | 0 |
| 3 | 700 | 0 | 24 |
| 4 | 400 | 0 | 24 |
| 5 | 150 | 3.15 | 0 |
| 6 | 400 | 0 | 16 |
| 7 | 500 | 0 | 16 |
| 8 | 750 | 0 | 20 |
| 9 | 450 | 2.1 | 0 |
| 10 | 200 | 0.72 | 0 |
| 11 | 300 | 3.15 | 0 |
| 12 | 400 | 0 | 24 |
| 13 | 1400 | 0 | 0 |
| 14 | 300 | 0 | 0 |
| 15 | 600 | 0 | 6 |
| 16 | 0 | 0 | 20 |
| 17 | 450 | 0 | 14 |
| 18 | 0 | 1.75 | 0 |
| 19 | 300 | 0.32 | 0 |
| 20 | 600 | 0.54 | 0 |
| 21 | 900 | 0 | 0 |
| 22 | 250 | 4.5 | 0 |
| 23 | 0 | 1.08 | 0 |
| 24 | 700 | 4.5 | 0 |
| 25 | 150 | 0 | 12 |
