# Supplementary material for: Targeting impulsivity in Parkinson’s disease using atomoxetine
Source: Brain. 2014 Jun 3;137(7):1986–97. doi: 10.1093/brain/awu117 (PMC4065022; doi:10.1093/brain/awu117)
Supplement: Supplementary Data [file supp_awu117_suppl_data.zip › brain-2013-02050-File011.docx]

Supplementary Table 2. Summary of behavioural measures at placebo baseline for patients receiving a DA agonist and those not receiving this class of drug. There were no significant differences on any measure. Data represent mean (SEM) values.

| **Measure** | **DA agonist subgroup** | |
| --- | --- | --- |
|  | **Yes** | **No** |
| **Stop Signal Task**  Successful stops (%)  Median Go RT (ms)  SSRT (ms) | **N=18** | **N=4** |
|  | 49.7 (2.3) | 45.6 (3.4) |
|  | 428 (15) | 498 (53) |
|  | 209 (17) | 226 (25) |
| SSD | 200 (24) | 219 (64) |
| **Cambridge Gamble Task** | **N=18** | **N=4** |
| Deliberation time | 2544 (162) | 3177 (476) |
| Proportion bet | 58.6 (3.4) | 51.1 (7.7) |
| Risk adjustment | 1.09 (0.2) | 0.69 (0.16) |
| Delay aversion | 0.23 (0.05) | 0.28 (0.12) |
| **Information Sampling Task** | **N=16** | **N=2** |
| Number of boxes opened | 12.4 (1.7) | 18.4 (6.5) |
| Box opening latency (log ms) | 3.06 (0.05) | 2.8 (0.2) |
| Decision latency (ms) | 4.18 (0.05) | 4.18 (0.07) |
| **One-Touch Stockings of Cambridge** | **N=18** | **N=4** |
| Problems solved on first choice | 3.16 (0.11) | 3.2 (0.21) |
| Latency to first choice (ms) | 23921 (1848) | 31021 (11143) |
| Latency to correct (ms) | 32869 (3408) | 44190 (13017) |
| **Rapid Visual Information Processing** | **N=18** | **N=4** |
| Mean latency (ms) | 507 (37) | 531 (86) |
| Hits | 15.39 (1.44) | 10.25 (3.94) |
| False alarms | 4.5 (1.6) | 3.5 (1.9) |
| A’ | 0.88 (0.02) | 0.83 (0.04) |
| B’ | 0.86 (0.04) | 0.84 (0.08) |
| **Digit Span** | **N=17** | **N=1** |
| Forward | 9.3 (0.05) | 7 |
| Backward | 7.06 (0.06) | 6 |
