# Supplementary material for: Targeting impulsivity in Parkinson’s disease using atomoxetine
Source: Brain. 2014 Jun 3;137(7):1986–97. doi: 10.1093/brain/awu117 (PMC4065022; doi:10.1093/brain/awu117)
Supplement: Supplementary Data [file supp_awu117_suppl_data.zip › brain-2013-02050-File012.docx]

Supplementary Figure 1

Illustration of individual patient performance on the Stop Signal Task (successful stops). The data points of the 4 individuals who were not on DA agonists are identified in red. Performance is examined a) at placebo baseline, b) on atomoxetine, c) in terms of the magnitude of the drug response compared to placebo.
