# Supplementary material for: Targeting impulsivity in Parkinson’s disease using atomoxetine
Source: Brain. 2014 Jun 3;137(7):1986–97. doi: 10.1093/brain/awu117 (PMC4065022; doi:10.1093/brain/awu117)
Supplement: Supplementary Data [file supp_awu117_suppl_data.zip › brain-2013-02050-File009.docx]

Supplementary material

In an exploratory post-hoc analysis, we addressed the effects of atomoxetine in those patients who were not receiving DA agonists (N=4) and the majority of the current patient sample who were receiving this class of drugs (N=19). We caution that as the study was not designed to test a hypothesis about the potential role of DA agonists, these analyses are insufficiently powered, precluding any inferences concerning their impact on these aspects of cognition and the effects of atomoxetine presented here.

DA agonist vs non-DA agonist treated groups on placebo

Notably, independent samples t-tests between subgroups (DA agonist vs no DA agonist) revealed no significant differences at placebo baseline on any of the measures for any of the tasks we report here (all n.s. at α= 0.05). Means are presented in Supplementary Table 2.

Effects of atomoxetine in the subgroup of patients not receiving a DA agonist

Subjecting the data to the same repeated measures ANOVA to address the effects of atomoxetine separately for the two subgroups revealed the following pattern. No effects of atomoxetine emerged as significant on any measure in the 4 individuals in the non-DA agonist treated subgroup, clearly reflecting insufficient power. There was no effect of drug on SST successful stops [F(1,2)= 0.996, P=0.42]. For CGT deliberation time, the drug x order interaction was not significant [F<1]. For CGT risk adjustment, there was a non-significant drug x order interaction [F(1,2)= 10.38, P= 0.08] which was impossible to decompose because there was only one patient who received atomoxetine on the first session versus three who received it on the second. For IST colour decision latency, the interaction seen in the main analysis was impossible to examine as there were only 3 patients who completed this task and all received atomoxetine on the second session. On the RVP, the drug x order interactions were not significant for either log latency [F<1] or sensitivity A’ [F(1,2)= 1.06, P= 0.41].

Effects of atomoxetine in patients receiving a DA agonist

Some but not all effects remained significant for the DA agonist treated subgroup in line with the reduction in the sample size. On the SST successful stops measure, there was no longer a drug effect [F(1,15)= 2.57, P= 0.13]. For CGT deliberation time, the drug x order interaction was no longer significant but only at trend level [F(1,14)= 4.33, P= 0.06], which when decomposed showed a significant effect for those patients who received atomoxetine on the first session [F(1,8)= 7.33, P= 0.03] but not on the second [F<1]. For CGT risk adjustment, the drug x order interaction was no longer significant [F(1,14)= 2.37, P= 0.15]. For the IST, and RVP, significant drug x order interactions remained: IST decision latency [F(1,13)= 7.27, P= 0.02], RVP log latency [F(1,16)= 5.29, P= 0.035] and RVP sensitivity A’ [F(1,16)= 8.49, P= 0.01].

In addition, we focus on the most notable finding of the effect of atomoxetine on SST successful stops. In Supplementary Figure 1, we identify the data points of the 4 individuals who were not on DA agonists (in red) a) at placebo baseline, b) on atomoxetine and c) in terms of the magnitude of their drug response compared to placebo. These patients did not differ significantly from the rest of the group. The magnitude of the drug response seen in Subject 1 falls within 2 SD of the mean (and as such, is not an outlier) but happens to be the largest out of the whole group for this measure. That is, a patient not receiving DA agonist showed the greatest improvement in inhibitory success. Were this datapoint to be considered in the framework of a hypothesis, it runs contra to that of a beneficial effect of atomoxetine just in those patients on DA agonists, or no effect in those not on DA agonists. However, we stress again that no inferences can be drawn on the basis of these few individual datapoints.
